# Supplementary material for: Investigation of the knowledge, attitudes, and perceptions regarding the utilization of rosemary among the population in Jordan
Source: PLoS One. 2024 Aug 26;19(8):e0307575. doi: 10.1371/journal.pone.0307575 (PMC11346737; doi:10.1371/journal.pone.0307575)
Supplement: S1 Appendix — (DOCX) [file pone.0307575.s001.docx]

**The Questionnaire**

**The Digital Approval**

Dear participants

A group of researchers are conducting research to evaluate the Jordanian understanding of the uses of Rosemary.

Your participation is crucial for the success of this research. Note that your involvement with this research is voluntary, and there are no dangers in participating in it.

All of the information gathered for this research will remain secret and won’t be used outside of the research’s purpose.

This form will take you only 4 minutes to fill.

Note that by agreeing to this form you will agree to

1. You have read the information above
2. You have agreed voluntarily to participate in this research

The digital approval

I Agree

I Disagree

**Part A: Demographics**

Gender

- Female
- Male

Age: ---------------

Marital status

- Single
- Married
- Divorced
- Widow

Education level

- I did not complete my study
- High school certificate
- Diploma
- Bachelor's degree
- Postgraduate degree

Occupation

- Student
- Unemployed
- Employee in a health sector
- Employee in a non-health sector
- Academic
- Freelancers or business owner
- Retired
- Other: --------

Permanent place of residence

- North of Jordan (Irbid/ Jarash/ Ajloun/ Al-Mafraq)
- Centre of Jordan (Amman/ Al-Zarqa/ Al-Balqa/ Madaba)
- South of Jordan (Al-Karak/ Ma’an/ Al-Tafelah/ Al-Aqaba)

Monthly Income

- <250 JoD
- 250-500 JoD
- 500-750 JoD
- 750-1000 JoD
- >1000 JoD

Health Insurance Status

- Insured
- Uninsured

Do you use/smoke tobacco products (Cigarettes)?

- Yes
- No

Do you suffer from any chronic disease(s), such as hypertension, diabetes, etc.?

- Yes
- No

Do you take any chronic medications (anti-hypertensive, diabetic drugs, etc....)?

- Yes
- No

Have you ever used rosemary or rosemary oil for medical purposes?

- Yes
- No

**If yes:** **Part B: Attitude and Practice toward Rosemary and its oil**

What was the nature of the Rosemary used?

- Oil Yes No
- Leaves Yes No

How did you apply/use the Rosemary?

- Inhalation Yes No
- Orally Yes No
- Gently rubbed on the area Yes No

Where did you get the rosemary/oil that you used for medical purposes?

- I bought it from Herbalist
- I bought it from a vegetable market
- I grow it at home
- I have bought it online
- Others......

Did you use rosemary or rosemary oil to treat?

- Hypercholesterolemia Yes No
- Joints problem Yes No
- GIT problem such as spasms, ulcer, inflammation …etc. Yes No
- Skin Conditions Yes No
- Hair cosmetic Yes No
- Stress/Anxiety relief Yes No
- Memory Enhancement Yes No

Did rosemary or its oil help in relieving your health issue?

- Yes
- No

Who advised you regarding the use of rosemary or its oil?

- A doctor
- A pharmacist
- A nurse
- Family and friends
- I read about it on social media
- I heard about it on TV
- I read about it on medical journals
- I just decided to use it by myself

**Part C: Knowledge About the Medicinal Uses of Rosemary**

Do you believe in the healing power of plants?

- Strongly Agree
- Agree
- Neutral
- Disagree
- Strongly Disagree

**Part D: Perception of Community Towards the Use of Natural Products in General**

Based in your knowledge, which is true about Rosemary?

- Has antioxidant properties True False I don’t know
- Has antibacterial properties True False I don’t know
- Can not be used as a natural aroma True False I don’t know
- Has antifungal properties True False I don’t know
- Has anti-inflammatory properties True False I don’t know
- Can be used as a natural preservative True False I don’t know
- Can not be used as a fragrance True False I don’t know
- Can not be used a condiment for food True False I don’t know

If you want to learn about the herbal products you want to use, what would be the source?

- Family or relatives Yes No
- Magazines Yes No
- Television Yes No
- Internet Yes No
- Physician Yes No
- Pharmacist Yes No
- Herbalist Yes No
- The Google form used (Arabic version):

<https://docs.google.com/forms/d/e/1FAIpQLSeJrdqxvzaV3yU_MUBvCupMXj-oRHRwTHHYLPZ3Kq0iOtAedA/viewform>
