# Supplementary figures and images for: Investigation of the knowledge, attitudes, and perceptions regarding the utilization of rosemary among the population in Jordan
Source: PLoS One. 2024 Aug 26;19(8):e0307575. doi: 10.1371/journal.pone.0307575 (PMC11346737; doi:10.1371/journal.pone.0307575)

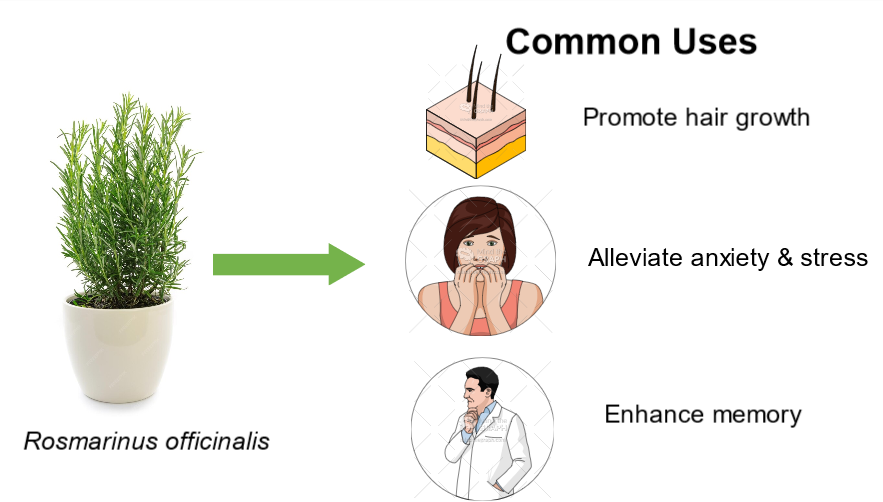

Supplement: S1 Graphical abstract — (TIF) [file pone.0307575.s002.tif]
